# Precise Prediction of Long-Term Urinary Incontinence after Robot-Assisted Laparoscopic Radical Prostatectomy by Readily Accessible “Everyday” Diagnostics during Post-Surgical Hospitalization

**DOI:** 10.3390/clinpract14030053

**Published:** 2024-04-23

**Authors:** Mirjam Naomi Mohr, Hannah Maria Ploeger, Marianne Leitsmann, Conrad Leitsmann, Fabian Alexander Gayer, Lutz Trojan, Mathias Reichert

**Affiliations:** 1 Department of Urology, University Medical Center Goettingen, 37075 Göttingen, Germany; mirjamnaomi.mohr@med.uni-goettingen.de (M.N.M.);; 2 Department of Pediatrics, University Hospital Bonn, 53127 Bonn, Germany; 3 Department of Urology, Medical University Graz, 8010 Graz, Austria

**Keywords:** robot-assisted laparoscopic radical prostatectomy, urinary incontinence, pad test, pad changing, nerve sparing, EPIC-26

## Abstract

**Aim and Objectives:** We aimed to test the predictive value of readily accessible and easily performed post-surgical “bedside tests” on their validity of long-term urinary incontinence (UI) (≥12 months) in patients following robot-assisted laparoscopic radical prostatectomy (RALP). **Material and Methods:** Patients undergoing RALP between July 2020 and March 2021 were prospectively included and subdivided into two groups based on their pad usage after 12 months (0 vs. ≥1 pad). After catheter removal, patients performed a 1 h pad test, documented the need for pad change in a micturition protocol and received post-voiding residual urine volume ultrasound. Univariate and multivariable analyses were used to demonstrate the predictive value of easily accessible tests applied after catheter removal for UI following RALP. **Results:** Of 109 patients, 47 (43%) had to use at least one pad (vs. 62 (57%) zero pads) after 12 months. Univariate testing showed a significant difference in urine loss between both groups evaluated by the 1 h pad test performed within 24 h after catheter removal (70% < 10 mL, vs. 30% ≥ 10 mL, *p* = 0.004) and in the need for pad change within the first 24 h after catheter removal (14% dry pads vs. 86% wet pads, *p* = 0.003). In multivariable analyses, the combination of both tests (synoptical incontinence score) could be confirmed as an independent predictor for UI after 12 months (*p* = 0.011). **Conclusions:** Readily accessible “everyday” diagnostics (pad test/change of pads after catheter removal) following RALP seem to be associated with a higher rate of long-term UI. This finding is crucial since patients with a potentially higher need for patient education and counselling can be identified using these readily accessible tests. This could lead to a higher patient satisfaction and improved outcomes.

## 1. Introduction

Urinary incontinence (UI) after robot-assisted laparoscopic radical prostatectomy (RALP) is a feared side effect after potentially curative surgical treatment of prostate cancer (PCa) [1,2].

Multiple studies focused on the identification of pre-, intra-, and postoperative parameters to predict long-term UI [3,4] or recovery of early urinary continence [5,6,7]. Focus thus far has been placed mostly on anatomical-, surgery-, and patient-related risk factors to predict UI. Even expensive and not broadly available diagnostics, like magnetic resonance imaging (MRI) of the pelvic floor, have been performed to demonstrate the correlation of anatomic features with a higher risk of post-prostatectomy incontinence (PPI) [6]. Hereby, the length of the membranous urethra (MUL) seems to be a predictive parameter for PPI. In their systematic review, Mac Curtain et al. even suggested establishing the MRI as a standard diagnostic tool after the diagnosis of PCa in order to measure the MUL and guide treatment decisions [8]. In addition to MUL, age and prostate gland volume are proven patient-related risk factors for PPI [9,10,11].

Aside from anatomical risk factors, changes in the urethral function after surgery further influence the risk of developing PPI [12]. Nevertheless, intraoperative testing of the sphincter function did not seem to have a predictive value [13].

Surgery-related risk factors for PPI, such as damage to the external (striated) urethral sphincter, should also be considered. Skeldon et al. developed a so-called “SM score” (striated muscle score), which postulates that the occurrence of PPI rises with an increasing amount of apical striated muscle tissue in the prostatectomy specimen [14]. The preservation, reconstruction, and reinforcement of the anatomic structures in the pelvis could have a positive impact on the continence after RALP [15]; e.g., the preservation of the full functional urethral length correlates with a better post-prostatectomy continence [7,16]. Additionally, preserving or reconstructing the bladder neck seems to correlate with higher continence rates [10,15].

The disadvantage of the abovementioned pre-, intra- and postoperative diagnostics and parameters for PPI lies in their difficult access and their high cost. Especially nowadays with the expected upheaval in health care, implementing those procedures into everyday clinical practice is not feasible.

On the other hand, the advantages of identifying individual predictors for PPI, even if they are collected postoperatively, are intriguing. In this way, patients with a higher risk of postoperative incontinence after RALP could be offered better, closer, and more individual care in the immediate postoperative course.

Our goal for this study was to find a readily accessible tool to predict long-term PPI. Therefore, we prospectively evaluated our standardized postoperative continence examinations regarding their predictive value on long-term UI after RALP.

## 2. Materials and Methods

This is a single-center, prospective evaluation of patients who underwent RALP as a curative treatment for PCa in the Department of Urology at the University Medical Center Goettingen (UMG, Göttingen, Germany) between July 2020 and March 2021.

After approval of the study by the local ethics committee of the Medical Association of Berlin (Eth-12/16), the Prostate Cancer Center of the UMG, certified by the German Cancer Society [17], began participating in the ongoing prospective, population-based Prostate Cancer Outcomes (PCO) study (DRKS00010774) [18]. The ethics committee of the UMG approved the amendment of this study as part of the PCO study (Eth-40/3/19Ü, approval date: 1 February 2022).

### 2.1. Study Population

Patients who underwent RALP as a curative treatment for histologically confirmed localized PCa in the Department of Urology at the UMG between July 2020 and March 2021 were prospectively included. The staging was carried out according to current guidelines (German-S3-Guidelines, European Association of Urology Guidelines) [19,20].

Patients were excluded if they received other local therapies like radiation, performed watchful waiting or active surveillance, or were receiving multimodal therapy for locally advanced PCa. Patients with neurological diseases like neurological bladder disorder in their anamnesis were also excluded as well as those patients with the inability to answer questionnaires.

### 2.2. Surgical Technique

All RALPs were performed by one of three surgeons using the DaVinci SI^©^ system (Intuitive Surgica. Sunnyvale, CA, USA). All surgeons had an experience of at least more than 450 RALPs each. The surgical techniques, e.g., preservation and reconstruction of the pelvic floor, were standardized (e.g., Rocco Stitch [21], apical dissection while preserving the functional length of the urethral sphincter as described by Schlomm et al. [22], etc. Organ-limited disease (≤cT2) was evaluated by digital rectal examination. Preservation of the neurovascular bundle was performed whenever the oncological option with respect to the guidelines was given and when it was in agreement with the patients wish. Intrafascial nerve sparing (NS) approach as described by Budäus et al. for the open approach [23] was routinely used. For oncological safety, the NeuroSAFE frozen section technique (Neurovascular structure-adjacent frozen-section examination) [24] was performed in case of a NS approach. If there was a cancer-positive area, the corresponding bundle was fully resected. Histopathological margins in the final specimen were defined as R0, R1 and R2 depending on the amount of cancer in the margin. NS was categorized into “no NS”, “unilateral NS” and “bilateral NS”.

A transurethral catheter was placed in all patients after surgery and remained until a sufficient cystogram/retrograde urethrocystography. The radiological control usually took place 5 to 7 days post-surgery. The duration of the catheter was dichotomized between ≤7 days and ≥8 days. After removal of the catheter, patients received standardized instructions by a physician to train the pelvic floor (Kegel exercise). They remained hospitalized for at least 24 h after catheter removal. Usually within 5 h after catheter removal, the patients were trained again by physiotherapists.

### 2.3. Data Collection and Outcomes

Data collection took place right before and after surgery as well as one year after RALP. In addition to the physical examination, patients were asked to answer the questions of the fifth version of the EPIC-26 in addition to standardized questionnaires like IPSS (international prostate symptom score) and ICIQ (international consultation of continence questionnaire).

Patients stayed in the hospital about one week after surgery until the removal of their catheter. Patients recorded their voiding conditions for 24 h (voiding rates, amount per fraction, pad usage and indication for changing pad) after the catheter was removed. The day after the removal, patients performed the standardized 1 h pad test [25]. Before hospital discharge, post-voiding residual urine volume (PVR) was measured by ultrasonography.

We categorized the postoperative 1 h pad test result according to the loss of urine: <10 mL (“good”) or ≥10 mL (“bad”). The need of pad change was categorized into changing the still dry pad, i.e., for hygienic reasons (category: “dry”) vs. changing the pad due to wetness, no matter how moist (category: “wet”) (evaluated by patient). When patients were categorized “bad” or “wet” in at least one of the two “bedside tests”, they were categorized “early incontinent” in a so-called synoptical incontinence score. Micturition volume was dichotomized by the amount of urine (≥100 mL vs. <100 mL) as well as PVR (≥30 mL vs. <30 mL).

The 12-month follow-up consisted of the EPIC-26 questionnaire. Answers given by the patients were scored according to the standardized scoring instructions [26]. The EPIC-26 questionnaire consists of the following 5 domains: UI, urinary irritative/obstructive symptoms, hormonal function, gastrointestinal symptoms, and sexual function. All domains have a point range from 0 to 100, with less points indicating lower function.

We categorized patients according to their 12-month follow-up. Those with 0 pads per day were defined as “continent” and ≥1 pad per day as “incontinent”.

The primary endpoint of this study was the continence status 12 months after RALP evaluated by pad usage. Secondary outcomes were patient- and surgery-related risk factors for PPI.

### 2.4. Statistical Analysis

Categorical variables were described using numbers and percentages; continuous variables were described using mean and standard deviation (SD). Dichotomous outcomes were evaluated using univariate and multivariable logistic regression models. The normality of continuous variables was tested with the Q-Q Plot, and in case of confirmation, statistically analyses were performed using the *t*-test. Non-normal distributions were analyzed using the Wilcoxon rank-sum test. Statistical significance was set at *p* < 0.05. All *p*-values were 2-sided. Variables were considered for inclusion in the multivariable model based on statistical significance (*p* < 0.1) from univariate logistic regression analyses and based on their literature-based relevance as potential confounders. They retained in the final multivariable model if *p* < 0.05. Covariables consisted of prostate volume (≤40 vs. >40 mL), NS (bilateral NS vs. unilateral NS vs. no NS), incontinence score (early incontinent vs. early continent) as well as BMI and age as continuous variables. The final multivariable logistic regression model was assessed for goodness of fit (calibration) with the Hosmer–Lemeshow test [27]. All statistical analyses were performed using IBM SPSS Statistics version 29.0.0.0.

## 3. Results

Between July 2020 and March 2021, we performed 148 RALPs. For statistical evaluation, 109 patients were considered as they had a complete set of follow-up data 12 months after surgery.

Patients’ characteristics are shown in Table 1; tumor characteristics including histopathological findings in the prostatectomy specimen as well as their surgical approach are shown in Table 2. According to their pad usage 12 months after surgery 47 (43%), patients were further defined as “incontinent”, while 62 patients could be defined as “continent” (57%).

A significant difference could be found in regard to age with younger patients being more likely to be continent than older patients (*p* = 0.03) and in the prostate volume (*p* = 0.01). The BMI had no significant influence in univariate analysis on continence after 12 months (*p* = 0.7).

While iPSA (*p* = 0.9), pN (*p* = 0.2), and the R status (*p* = 0.2) were not significant, pT (*p* = 0.01) and GG ISUP (*p* = 0.02) showed a significant difference between both groups, with a higher rate of incontinence correlating with increasing local advancement of the PCa. NS showed a significant influence on the continence status after 12 months.

Table 3 shows the pre- and postoperative voiding conditions of the total cohort and subdivided according to the continence status 12 months after RALP.

Preoperative voiding conditions (IPSS (*p* = 0.4) and ICIQ (*p* = 0.6)) as well as postoperative voiding conditions (micturition volume (*p* = 0.4) and PVR (*p* = 0.8)) did not have any predictive value for the pad usage 12 months after surgery.

Table 4, Table 5 and Table 6 include the easily performed post-surgical “bedside tests” and their influence on long-term UI.

We saw a highly significant correlation (*p* = 0.004) between a “bad” postoperative pad test and the need for pad usage after 12 months (see Table 4). The need for changing pads due to wetness after catheter removal within the initial 24 h also significantly correlates with the pad usage after 12 months (see Table 5) as well as the synoptical incontinence score (*p* < 0.001) (see Table 6).

In the non-NS group, the pad test kept its predictive value (*p* = 0.018), while the pad changing did not stay significant (*p* = 0.5). In the NS group (uni- and bilateral), pad changing (*p* = 0.013) had a predictive value, but the pad test lost its significance (*p* = 0.2). Dividing between uni-and bilateral NS, the preservation of both neurovascular bundles led to significantly better continence rates than the preservation of just one neurovascular bundle (*p* = 0.001).

The multivariable analysis evaluating predictors for continence status after 12 months confirmed prostate volume (*p* = 0.003), NS (*p* = 0.020) and the established incontinence score (*p* = 0.011) as significant predictors (see Table 7). Goodness-of-fit was assessed using the Hosmer–Lemeshow Test [27], indicating a good model fit, χ^2^ (8) = 12.216, *p* > 0.05.

## 4. Discussion

This study aimed to evaluate the predictability of readily accessible “everyday” diagnostic tests performed after RALP during hospitalization on 12 months continence outcome. To do so, we prospectively evaluated our standardly performed tests after catheter removal on days 5–7 after RALP. Our results show that readily accessible postoperative tests seem to predict long-term UI. In the presented patient population, the need for pad changing within the first 24 h and the pad test showed highly significant correlations with long-term UI (*p* = 0.003, *p* = 0.004, respectively) as well as the synoptical incontinence score (*p* < 0.001).

The main focus of this study was not to identify modifiable risk factors for UI or to examine parameters causing UI to eventually be able to intervene circumstances that lead to less favorable continence status. This study should demonstrate that patients can easily be separated after surgery by everyday diagnostics during hospitalization after RALP in regard to UI. In the multivariable analyses, the predictive value of the “bedside tests” could be confirmed and the synoptical incontinence score was an independent predictor for UI after 12 months (*p* = 0.01). The 1 h pad test, as well as the need for pad changing within the first 24 h combined as the synoptical incontinence score is readily accessible and can easily be implemented into everyday clinical life without raising investments of time or costs. In our knowledge, this is the first developed “bedside-test” score to predict UI that can be easily adopted.

In our cohort, we identified 47 “incontinent” patients applying the definition of one or more pads per day and 62 “continent” patients (0 pads per day) after 12 months. Up to date, there is no agreement about a cut-off value for pad usage to define satisfying post-surgery urinary continence [28]. We defined the cut-off for incontinence as one or more pads per day, because we agree that the necessity of using even one (safety) pad can significantly reduce the patients’ quality of life [29]. In concordance, Cortes et al. described urinary continence as absolutely no pad use with the use of even 1 safety pad not fulfilling the definition of classical urinary continence [30]. Improvements in surgical approaches with the implementation of new surgical standards (preserving full functional urethral length, NeuroSAFE, bladder neck preservation and reconstruction, etc.) and scientific understanding for PPI now lead to higher rates of urinary continence. Unfortunately, most studie to date still define continence as 0–1 pads per day.

Concerning risk factors for incontinence, we observed an important difference in the age at time of surgery between both patient populations. The “incontinent” patient population had a significantly older age (mean: 67 years) compared to the “continent” patients (mean: 64 years) (*p* = 0.03). Age is a proven patient-sided risk factor for PPI. One of the most recent studies by Cano Garcia et al. divided their patient population into three age groups (≤60 years, 61–69 years, and ≥70 years). They saw significant differences between these three groups in terms of long-term urinary continence (90% vs. 84% vs. 69% for, respectively, group 1 vs. 2 vs. 3; *p* = 0.018). The younger age groups (Group 1 (odds ratio (OR) 4.73, 95% CI 1.44–18.65, *p* = 0.015) and 2 (OR 2.94, 95% CI 1.23–7.29; *p* = 0.017)) were independent predictors for urinary continence [7].

Further on, we did not see any correlation between preoperative assessments for lower urinary tract symptoms (LUTS) (like IPSS (*p* = 0.4) and ICIQ (*p* = 0.6)) and postoperative UI. In their review, Lardas et al. described the same results, postulating that “there is a lack of data to draw any conclusions about the role of IPSS” [31].

Surgical outcome regarding NS had a significant influence on postoperative UI in our population, as we divided all procedures/patients into three groups. Group 1 reached a successful bilateral NS (*n* = 29) with a majority of patients (*n* = 24, 83%) being continent after 12 months (no pad usage), whereas patients with unilateral NS, Group 2 (*n* = 31), and no NS, Group 3 (*n* = 49), showed nearly equal continence and incontinence rates (42% continent (Group 2), 51% continent (Group 3), respectively) (*p* = 0.03). Dividing between uni-and bilateral NS, the preservation of both neurovascular bundles leads to significantly better continence rates than the preservation of just one neurovascular bundle (*p* = 0.001).

It has been shown that NS RALPs are associated with a better functional outcome overall (continence and erectile function) [32]. With our results, we agree that the preservation of both neurovascular bundles seems to be the most favorable situation for long-term continence [33].

Finally, we saw significantly advanced tumor characteristics in the “incontinent” patient population with worse pT status and GG ISUP (*p* = 0.01 and *p* = 0.02, respectively). Interestingly, iPSA, Gleason score, lymph nodes and resection status did not differ between the “continent” and “incontinent” patients. However, there is a lack of evidence about the role of preoperative PSA, Gleason score, and pT status for the development of postoperative UI, as Lardas et al. discussed in their review about patient- and tumor-related prognostic factors [31]. Our study mainly agrees with these results with exception of the pT status.

Interestingly, micturition volume (*p* = 0.4) and PVR (*p* = 0.8), possible indicators of a very narrow bladder neck, did not seem to predict UI or a better postoperative continence, as would have been expected. The possible influence of micturition volume or PVR after micturition has not been considered by many studies thus far. Takeshima et al. showed that a decrease in micturition volume from pre- to postoperative testing is associated with worsening postoperative incontinence [34]. But solely postoperative micturition volume has not been part of research so far and is dependent on multiple other circumstances like gland volume, age and other “LUTS-causing” factors.

To summarize the results discussed above, we see a patient population and UI outcome that is consistent with results published to date. Therefore, our findings on predicting long-term UI can easily be adopted to an “every day clinical patient population”.

We consider the availability of easily performed and easy to implement tools in everyday clinical life to predict long-term UI after RALP to be crucial. To perform a standardized 1 h pad test the day of catheter removal and reporting of pad usage within the first 24 h after catheter removal is not expected to overwhelm patients and does not involve any higher costs. Patients at risk for long-term postoperative UI should have the chance to be offered more intense postoperative counselling and care, like physiological education, pelvic floor exercises etc., to minimize the risk of having to use pads 12 months after surgery.

Cofounders of this study show that surgical techniques, like preserving both neurovascular bundles, should be generously performed under consideration of the oncological safety. We standardly perform intrafascial NS RALPs under whole mount frozen section control (NeuroSAFE). This procedure does not compromise oncological safety but leads to less secondary resections with a better functional outcome in regard to erectile function and UI [24].

### Strength and Limitations

The main limitation of this study is its population size, since this might affect the generalizability of the findings. Further multicenter studies with a larger population size are needed to confirm the findings and improve the statistical power. Long-term incontinence was evaluated after 12 months. Continence can recover even between 12 and 24 months post-RALP [35] even though most changes occur within the first 12 months. Larger population sizes and a longer follow-up are needed in the future.

The biggest advantage of this study is its clinical benefit without the necessity of using more resources. Its prospective character in combination with the usage of the EPIC-26 as a patient-reported outcome measure is an additional strength of this study. In addition to the limitation of being a unicentric study, the unicentric data collection has the advantage of highly standardized procedures. Every surgery is performed the same way. Pre-, peri- and postoperative data acquisition was also performed in a standardized manner without disturbance of changing centers.

Furthermore, we highly believe that it is antiquated to draw the line in the evaluation between long-term incontinent and continent patients at >1 safety pad. Continence should be defined as no pad usage within 24 h. The aim of today’s RALPs should be the total absence of pad usage by patients, since even one pad can cause a severe reduction in quality of life.

## 5. Conclusions

A pad test with ≥10 mL loss of urine on the day of catheter removal and the need for pad changing within the first 24 h after catheter removal following RALP seem to be associated with a higher rate of long-term UI (12 months). This finding is crucial, since with these readily accessible and easily performed tests, patients with a potentially higher need for patient education and counselling can be identified—without the necessity of using more resources. Patients at risk for long-term postoperative UI should have the chance to be offered more intense postoperative care, like physiological education, pelvic floor exercises, etc., to minimize the risk of pad usage 12 months after surgery. This could lead to a higher patient satisfaction, better patient treatment and outcomes.

## Figures and Tables

**Table 1:** Patient characteristics of the total cohort and subdivided according to the continence status after 12 months.

		Total *n* = 109	Continent *n* = 62 (57%)	Incontinent *n* = 47 (43%)	*p*-Value
Age [years]	Mean (SD)	66 (6.2)	64 (6.6)	67 (5.4)	0.03
BMI [kg/m^2^]	BMI < 24	13 (12%)	6 (46%)	7 (54%)	0.7
	BMI 24–<30	72 (67%)	45 (63%)	27 (37%)	
	BMI 30–<35	19 (17%)	10 (53%)	9 (47%)	
	BMI ≥ 35	4 (4%)	1 (25%)	3 (75%)	
Prostate volume [mL]	≤40	50 (46%)	22 (44%)	28 (56%)	0.01
	>40	59 (54%)	40 (68%)	19 (32%)	
Catheterization time (after RALP)	≤7 days	97 (89%)	53 (55%)	44 (45%)	0.2
	≥8 days	12 (11%)	9 (75%)	3 (25%)	

(SD = standard deviation, BMI = Body Mass Index, kg/m^2^ = kilogram per square meter, mL = milliliter, RALP = robot-assisted laparoscopic radical prostatectomy).

**Table 2:** Tumor characteristics of the total cohort and subdivided according to the continence status after 12 months.

		Total *n* = 109	Continent *n* = 62 (57%)	Incontinent *n* = 47 (43%)	*p*-Value
iPSA [ng/mL]	<10	77 (71%)	43 (56%)	34 (44%)	0.9
	10–<20	20 (18%)	15 (75%)	5 (25%)	
	≥20	12 (11%)	4 (33%)	8 (67%)	
GG ISUP	1	3 (3%)	2 (67%)	1 (33%)	0.02
	2	55 (51%)	38 (69%)	17 (31%)	
	3	24 (22%)	12 (50%)	12 (50%)	
	4	10 (9%)	1 (10%)	9 (90%)	
	5	16 (15%)	9 (56%)	7 (44%)	
pT status	pT2	64 (59%)	43 (67%)	21 (33%)	0.01
	pT3-4	45 (41%)	19 (42%)	26 (58%)	
pN status	pN0	102 (94%)	59 (58%)	43 (42%)	0.2
	pN1	6 (6%)	2 (33%)	4 (67%)	
pPn	0	27 (27%)	20 (74%)	7 (26%)	0.04
	1	73 (73%)	37 (51%)	36 (49%)	
R status	R0	81 (75%)	49 (60%)	32 (40%)	0.2
	R1	26 (24%)	13 (50%)	13 (50%)	
	R2	1 (1%)	0	1 (100%)	
GS	6	3 (3%)	2 (67%)	1 (33%)	0.1
	7	81 (75%)	50 (62%)	31 (38%)	
	8	8 (7%)	1 (13%)	7 (87%)	
	9	16 (15%)	9 (56%)	7 (44%)	
NS	Bilateral NS	29 (27%)	24 (83%)	5 (17%)	0.03
	Unilateral NS	31 (28%)	13 (42%)	18 (58%)	
	No NS	49 (45%)	25 (51%)	24 (49%)	

(iPSA = initial prostate-specific antigen, ng = nanogram, mL = milliliter, GG ISUP = ISUP grade group system for prostate cancer, GS = Gleason score, NS = nerve sparing).

**Table 3:** Pre- and postoperative voiding conditions of the total cohort and subdivided according to the continence status after 12 months.

		Total *n* = 109	Continent *n* = 62 (57%)	Incontinent *n* = 47 (43%)	*p*-Value
IPSS (preoperative)	<8	58 (55%)	35 (60%)	23 (40%)	0.4
	8–19	42 (40%)	24 (57%)	18 (43%)	
	20–35	6 (5%)	2 (33%)	4 (67%)	
ICIQ (preoperative)	No incon.	72 (72%)	41 (57%)	31 (43%)	0.6
	Light incon.	19 (19%)	10 (53%)	9 (47%)	
	Mid incon.	5 (5%)	4 (80%)	1 (20%)	
	Severe incon.	5 (5%)	1 (20%)	4 (80%)	
Micturition volume [mL] (postoperative)	<100	39 (51%)	25 (64%)	14 (36%)	0.4
	≥100	38 (49%)	21 (55%)	17 (45%)	
PVR [mL] (postoperative)	<30	77 (78%)	44 (57%)	33 (43%)	0.8
	≥30	22 (22%)	12 (55%)	10 (45%)	

(IPSS = international prostate symptom score, ICIQ = international consultation of continence questionnaire, incon. = incontinence, mL = milliliter, PVR = post voiding residual urine volume).

**Table 4:** Postoperative pad test of the total cohort and subdivided according to the continence status after 12 months.

		Total *n* = 81	Continent *n* = 47 (58%)	Incontinent *n* = 34 (42%)	*p*-Value
Pad test	Good	57 (70%)	39 (68%)	18 (32%)	0.004
	Bad	24 (30%)	8 (33%)	16 (67%)	

**Table 5:** The need for changing pads the first 24 h after catheter removal of the total cohort and subdivided according to the continence status after 12 months.

		Total *n* = 74	Continent *n* = 42 (57%)	Incontinent *n* = 32 (43%)	*p*-Value
Pad changing	Dry	10 (14%)	10 (100%)	0	0.003
	Wet	64 (86%)	32 (50%)	32 (50%)	

**Table 6:** The incontinence score of the total cohort and subdivided according to the continence status after 12 months.

		Total *n* = 91	Continent *n* = 54 (59%)	Incontinent *n* = 37 (41%)	*p*-Value
Incontinence score	Early continent	21 (23%)	19 (90%)	2 (10%)	<0.001
	Early incontinent	70 (77%)	35 (50%)	35 (50%)	

**Table 7:** Multivariable logistic regression model for continence status after 12 months.

		OR	95% Confidence Interval	*p*-Value
Age [years]	continuous	1.10	0.99–1.21	0.078
BMI [kg/m^2^]	continuous	1.14	0.98–1.32	0.100
Prostate volume [mL]	>40 (ref)			
	≤40	5.49	1.80–16.75	0.003
NS	Bilateral NS (ref)			0.061
	Unilateral NS	3.99	0.95–16.72	0.059
	No NS	6.15	1.33–28.40	0.020
Incontinence score	Early continent (ref)			
	Early incontinent	10.20	1.69–61.64	0.011

(OR = odds ratio, BMI = Body Mass Index, kg/m^2^ = kilogram per square meter, mL = milliliter, ref = reference, NS = nerve sparing).

## Data Availability

The data presented in this study are available on request from the corresponding author.

## References

[1] Palmer M.H., Fogarty L.A., Somerfield M.R., Powel L.L. (2003). Incontinence after prostatectomy: Coping with incontinence after prostate cancer surgery. Oncol. Nurs. Forum..

[2] Geraghty K., Keane K., Davis N. (2024). Systematic review on urinary continence rates after robot-assisted laparoscopic radical prostatectomy. Ir. J. Med. Sci..

[3] Oefelein M.G. (2004). Prospective predictors of urinary continence after anatomical radical retropubic prostatectomy: A multivariate analysis. World J. Urol..

[4] Van Kampen M., De Weerdt W., Van Poppel H., Feys H., Castell Campesino A., Stragier J., Baert L. (1998). Prediction of urinary continence following radical prostatectomy. Urol. Int..

[5] Nitta M., Tazawa M., Takahashi K., Naruse J., Oda K., Kano T., Uchida T., Umemoto T., Ogawa T., Kawamura Y. (2023). Variations in predictors for urinary continence recovery at different time periods following robot-assisted radical prostatectomy. Asian J. Endosc. Surg..

[6] Mao H., Zhang F., Zhang Z.Y., Yan Y., Hao Y.C., Huang Y., Ma L.L., Chu H.L., Zhang S.D. (2023). Predictive model of early urinary continence recovery based on prostate gland MRI parameters after laparoscopic radical prostatectomy. Beijing Da Xue Xue Bao Yi Xue Ban.

[7] Ando S., Sugihara T., Hinotsu S., Kishino H., Hirata D., Watanabe R., Yanase A., Yokoyama H., Hoshina H., Endo K. (2024). Early recovery of urinary continence after robot-assisted radical prostatectomy is associated with membranous urethra and neurovascular bundle preservation. Int. J. Urol..

[8] Mac Curtain B.M., Sugrue D.D., Qian W., O’Callaghan M., Davis N.F. (2023). Membranous urethral length and urinary incontinence following robot-assisted radical prostatectomy: A systematic review and meta-analysis. BJU Int..

[9] Cano Garcia C., Wenzel M., Humke C., Wittler C., Dislich J., Incesu R.B., Köllermann J., Steuber T., Graefen M., Tilki D. (2023). Impact of Age on Long-Term Urinary Continence after Robotic-Assisted Radical Prostatectomy. Medicina.

[10] Rajih E., Meskawi M., Alenizi A.M., Zorn K.C., Alnazari M., Zanaty M., Alhathal N., El-Hakim A. (2019). Perioperative predictors for post-prostatectomy urinary incontinence in prostate cancer patients following robotic-assisted radical prostatectomy: Long-term results of a Canadian prospective cohort. Can. Urol. Assoc. J..

[11] Heesakkers J., Farag F., Bauer R.M., Sandhu J., De Ridder D., Stenzl A. (2017). Pathophysiology and Contributing Factors in Postprostatectomy Incontinence: A Review. Eur. Urol..

[12] Dubbelman Y.D., Bosch J.L. (2013). Urethral sphincter function before and after radical prostatectomy: Systematic review of the prognostic value of various assessment techniques. Neurourol. Urodyn..

[13] Mohr M.N., Uhlig A., Strauß A., Leitsmann C., Ahyai S.A., Trojan L., Reichert M. (2023). Prospective evaluation of an intraoperative urodynamic stress test predicting urinary incontinence after robot-assisted laparoscopic radical prostatectomy. Urol. Ann..

[14] Skeldon S.C., Gani J., Evans A., Van Der Kwast T., Radomski S.B. (2014). Striated muscle in the prostatic apex: Does the amount in radical prostatectomy specimens predict postprostatectomy urinary incontinence?. Urology.

[15] Massanova M., Bada M., Crocetto F., Barone B., Arcaniolo D., Silvestri T., De Concilio B., Zeccolini G., Mazzon G., Celia A. (2020). Bowel suture technique for bladder neck reconstruction during RALP and its impact on early continence recovery. Minerva Urol. Nefrol..

[16] Hoeh B., Hohenhorst J.L., Wenzel M., Humke C., Preisser F., Wittler C., Brand M., Köllermann J., Steuber T., Graefen M. (2023). Full functional-length urethral sphincter- and neurovascular bundle preservation improves long-term continence rates after robotic-assisted radical prostatectomy. J. Robot. Surg..

[17] Kowalski C., Ferencz J., Albers P., Fichtner J., Wiegel T., Feick G., Wesselmann S. (2016). Quality assessment in prostate cancer centers certified by the German Cancer Society. World J. Urol..

[18] Kowalski C., Roth R., Carl G., Feick G., Oesterle A., Hinkel A., Steiner T., Brock M., Kaftan B., Borowitz R. (2020). A multicenter paper-based and web based system for collecting patient-reported outcome measures in patients undergoing local treatment for prostate cancer: First experiences. J. Patient Rep. Outcomes.

[19] Mottet N., van den Bergh R.C.N., Briers E., Van den Broeck T., Cumberbatch M.G., De Santis M., Fanti S., Fossati N., Gandaglia G., Gillessen S. EAU Guidelines Prostate Cancer. Edn. Presentedat the EAU Annual Congress Milan 2021 EAU Guidelines Office: Arnhem, The Netherlands, 2021. http://uroweb.org/guidelines/compilations-of-all-guidelines/.

[20] Leitlinienprogramm Onkologie (Deutsche Krebsgesellschaft, Deutsche Krebshilfe, AWMF): S3-Leitlinie Prostatakarzinom, Langversion 6.0, 2021. http://www.leitlinienprogramm-onkologie.de/leitlinien/prostatakarzinom/.

[21] Rocco F., Gadda F., Acquati P., Carmignani L., Favini P., Dell’Orto P., Ferruti M., Avogadro A., Casellato S., Grisotto M. (2001). Personal research: Reconstruction of the urethral striated sphincter. Arch. Ital. Urol. Androl..

[22] Schlomm T., Heinzer H., Steuber T., Salomon G., Engel O., Michl U., Haese A., Graefen M., Huland H. (2011). Full funtional-length urethral sphincter preservation during radical prostatectomy. Eur. Urol..

[23] Budäus L., Isbarn H., Schlomm T., Heinzer H., Haese A., Steuber T., Salomon G., Huland H., Graefen M. (2009). Current technique of open intrafascial nerve-sparing retropubic prostatectomy. Eur. Urol..

[24] Schlomm T., Tennstedt P., Huxhold C., Steuber T., Salomon G., Michl U., Heinzer H., Hansen J., Budäus L., Steurer S. (2012). Neurovascular structure-adjacent frozen-section examination (NeuroSAFE) increases nerve-sparing frequency and reduces positive surgical margins in open and robot-assisted laparoscopic radical prostatectomy: Experience after 11,069 consecutive patients. Eur. Urol..

[25] Klarskov P., Hald T. (1984). Reproducibility and reliability of urinary incontinence assessment with a 60 min test. Scand. J. Urol. Neph..

[26] Sanda M.G., Wei J.T., Litwin M.S. (2002). Scoring Instructions for the Expanded Prostate Cancer Index Composite Short form (EPIC-26).

[27] Hosmer D.W., Lemeshow S. (2001). Applied Logistic Regression.

[28] Tienza A., Graham P.L., Robles J.E., Diez-Caballero F., Rosell D., Pascual J.I., Patel M.I., Mungovan S.F. (2020). Daily Pad Usage Versus the International Consultation on Incontinence Questionnaire Short Form for Continence Assessment Following Radical Prostatectomy. Int. Neurourol. J..

[29] Cooperberg M.R., Master V.A., Carroll P.R. (2003). Health related quality of life significance of single pad urinary incontinence following radical prostatectomy. J. Urol..

[30] García Cortés Á., Colombás Vives J., Gutiérrez Castañé C., Chiva San Román S., Doménech López P., Ancizu Marckert F.J., Hevia Suárez M., Merino Narro I., Velis Campillo J.M., Guillén Grima F. (2021). What is the impact of post-radical prostatectomy urinary incontinence on everyday quality of life? Linking Pad usage and International Consultation on Incontinence Questionnaire Short-Form (ICIQ-SF) for a COMBined definition (PICOMB definition). Neurourol. Urodyn..

[31] Lardas M., Grivas N., Debray T.P.A., Zattoni F., Berridge C., Cumberbatch M., Van den Broeck T., Briers E., De Santis M., Farolfi A. (2022). Patient- and Tumour-related Prognostic Factors for Urinary Incontinence After Radical Prostatectomy for Nonmetastatic Prostate Cancer: A Systematic Review and Meta-analysis. Eur. Urol. Focus.

[32] van der Slot M.A., Remmers S., van Leenders G.J.L.H., Busstra M.B., Gan M., Klaver S., Rietbergen J.B.W., den Bakker M.A., Kweldam C.F., Bangma C.H. (2023). Anser Prostate Cancer Network. Urinary Incontinence and Sexual Function After the Introduction of NeuroSAFE in Radical Prostatectomy for Prostate Cancer. Eur. Urol. Focus.

[33] Steineck G., Bjartell A., Hugosson J., Axén E., Carlsson S., Stranne J., Wallerstedt A., Persson J., Wilderäng U., Thorsteinsdottir T. (2015). Degree of preservation of the neurovascular bundles during radical prostatectomy and urinary continence 1 year after surgery. Eur. Urol..

[34] Takeshima Y., Yamada Y., Takemura K., Kimura N., Hakozaki Y., Miyakawa J., Taguchi S., Akiyama Y., Sato Y., Kawai T. (2022). The association between the parameters of uroflowmetry and lower urinary tract symptoms in prostate cancer patients after robot-assisted radical prostatectomy. PLoS ONE.

[35] Sacco E., Prayer-Galetti T., Pinto F., Fracalanza S., Betto G., Pagano F., Artibani W. (2006). Urinary incontinence after radical prostatectomy: Incidence by definition, risk factors and temporal trend in a large series with a long-term follow-up. BJU Int..

